# Retraction: Alotaibi, M.O., et al. Morpho-Physiological and Proteomic Analyses of *Eucalyptus camaldulensis* as a Bioremediator in Copper-Polluted Soil in Saudi Arabia. *Plants* 2019, *8*, 43

**DOI:** 10.3390/plants9091201

**Published:** 2020-09-14

**Authors:** Modhi O. Alotaibi, Afrah E. Mohammed, Taghreed A. Almutairi, Mudawi M. Elobeid, Plants Editorial Office

**Affiliations:** 1 Department of Biology, Faculty of Science, Princess Nourah bent Abdul-Rahman University, Riyadh 11474, Saudi Arabia; mouotaebe@pnu.edu.sa (M.O.A.); Tag32-27@hotmail.com (T.A.A.); 2 Department of Silviculture, Faculty of Forestry, University of Khartoum, Khartoum North, Shambat 13314, Sudan; emudawi2828@hotmail.com; 3 MDPI, St. Alban-Anlage 66, 4052 Basel, Switzerland

The *Plants* Editorial Office has been made aware that there are partly inadequate materials in the published paper [1]. In this paper, some aspects of proteomics data were incorrectly presented in parts of materials and methods, as the complete proteomics protocols used in generating the data were not reported in the paper. Although the outcome and conclusions of the study were not affected, the changes to the methods were so substantial that it was decided that a correction would not be appropriate, and the paper will therefore be retracted. The retraction has been agreed with both the Editor-in-Chief of *Plants* and the authors of [1]. 

The *Plants* Editorial Office apologizes to the readers of *Plants* for any inconvenience caused. To ensure the addition of only high-quality scientific works to the field of scholarly communication, the paper [1] is retracted and shall be marked accordingly. MDPI is a member of the Committee on Publication Ethics (COPE) and takes the responsibility to enforce strict ethical policies and standards very seriously.
